# Enzymatic Prenylation of Proteins and Peptides: From Cysteine *S*‐Prenylation to Tryptophan‐Selective Biocatalysis

**DOI:** 10.1002/chem.70995

**Published:** 2026-04-10

**Authors:** Daisuke Fujinami, Hikari Ozawa, Sohei Ito

**Affiliations:** ^1^ Graduate School of Integrated Pharmaceutical and Nutritional Sciences University of Shizuoka Shizuoka Shizuoka Japan

**Keywords:** biocatalysis, lipidation, peptides, posttranslational modifications, prenyltransferases

## Abstract

Posttranslational prenylation endows polypeptides with lipophilic properties, facilitating essential biological functions such as membrane targeting and signaling. Unlike linear fatty acyl groups, the branched and unsaturated structure of isoprenoids introduces complex sterics and restricted conformational degrees of freedom. These unique features enable the fine‐tuning of membrane behaviors, including lateral partitioning, packing defects, and hydrophobic mismatches, which can dictate biological outcomes. Focusing on biocatalytic prenylation, this review summarizes eukaryotic, cyanobactin, and *trans*‐isoprenyl diphosphate synthase (*trans*‐IDS)‐like prenyltransferases. We examine how active‐site motifs govern specificity for both the amino acid acceptor and the donor isoprenoid, thereby enabling the use of longer‐chain variants and geometric isomers, as well as regioselective modification of tryptophan. Furthermore, we compare the biophysical properties of emerging tryptophan *C*‐prenylation with those of conventional cysteine *S*‐prenylation and linear fatty acylation, highlighting their distinct roles in modulating polypeptide function and behavior. Finally, we focus on applications of enzymatic prenylation in antimicrobial development and protein engineering, outlining future opportunities for AI‐guided design of prenylated peptides and proteins.

## Graphical abstract

This review highlights biocatalytic prenylation as a versatile strategy for tailoring the functional properties of peptides and proteins. By comparing branched isoprenoids with linear lipids, we illustrate how specific prenyl architectures modulate the behaviors of lipidated proteins within membrane environments. We specifically focus on *trans*‐isoprenyl diphosphate synthase (*trans*‐IDS)‐like prenyltransferases, which expand the chemical space by accepting diverse donor substrates and enabling regioselective tryptophan modification. These enzymatic tools provide a foundation for future efforts to tune biological functions, including antimicrobial activity, liquid–liquid phase separation, and subcellular localization.

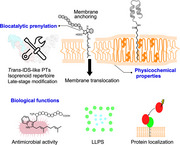

## 1 Introduction

**FIGURE 1:**
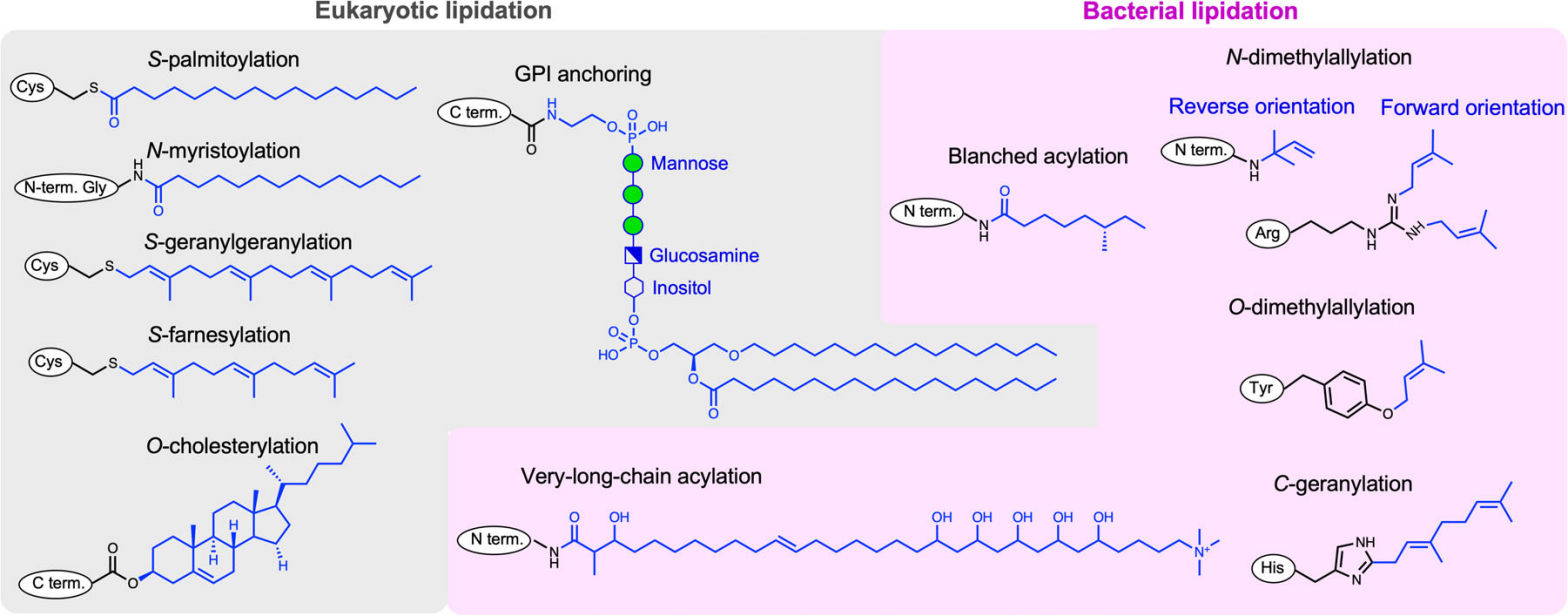
Major classes of protein and peptide lipidations. Lipid moieties are highlighted in blue. Modification sites, including specific amino acid residues and peptide termini, are indicated by white circles. The Figure illustrates the diversity of lipid modifications found in eukaryotes (left) and bacteria (right), spanning fatty acylation and various forms of prenylation.

The lipid bilayer serves as the fundamental architecture of life, creating a chemically unique interior and a vital interface with the environment [1]. Posttranslational protein lipidation occurs predominantly intracellularly, increasing protein hydrophobicity to drive membrane targeting and tune protein–membrane or protein–protein interactions, thereby regulating signaling at membrane interfaces [2]. In eukaryotes, the major classes of protein lipidation include fatty acylation (e.g., cysteine *S*‐palmitoylation and *N*‐terminal glycine *N*‐myristoylation), diacylglycerol‐based phosphoglyceride conjugation/anchoring (including GPI anchoring and phosphatidylethanolamine attachment), cholesterylation, and prenylation on cysteine residues (e.g., *S*‐farnesylation and *S*‐geranylgeranylation) [3, 4] (Figure 1). Even among lipid groups with comparable carbon counts, structural variations dictate distinct subcellular localizations (e.g., the plasma vs. organellar membranes), lateral partitioning (raft vs. nonraft domains), and membrane‐binding affinity [2, 5, 6, 7, 8]. Furthermore, postlipidation processing and interactions with binding partners can further refine these interactions; for instance, cysteine *S*‐prenylation is frequently followed by *C*‐terminal proteolysis and carboxyl methylation, and the prenyl groups may be shielded by specific solubilizing factors [9, 10, 11]. In archaea, the attachment of diphytanylglycerol diether (archaeol) to a cysteine residue within a *C*‐terminal lipobox has also been reported, and this lipidation contributes to protein localization to membranes [12].

In bacteria, lipidation extends beyond proteins to encompass peptides produced by nonribosomal peptide synthetase (NRPS) and ribosomally synthesized and posttranslationally modified peptide (RiPP) biosynthetic machineries [13, 14, 15, 16, 17]. These compounds form a class of secondary metabolites known as lipopeptides, featuring lipid moieties that are primarily fatty acyl chains but exhibit greater structural diversity than those in eukaryotic proteins. Beyond simple fatty acylation, these lipids can include very‐long‐chain fatty acids (e.g., > C30) [18], branched acyl tails [19], or fatty acids bearing functional groups such as hydroxyl, amino, dimethylguanidino, or trimethylammonium substituents [14, 16, 20] (Figure 1). Prenylation further expands this chemical space through distinct linkages to various heteroatoms or nucleophilic sites, including *C*‐prenylation (e.g., His and Trp) [21, 22, 23, 24, 25, 26], *N*‐prenylation (e.g., Trp, Arg, or the *N*‐terminal amino group) [27, 28, 29, 30], and *O*‐prenylation (e.g., Tyr, Thr, and Ser) [31, 32, 33, 34, 35, 36] (Figure 1). These modifications typically use prenyl chains shorter than the C15/C20 groups used in eukaryotic cysteine *S*‐prenylation [15, 37]. Significantly, lipopeptides exhibit potent biological activities, including antifungal, antimicrobial, and antineoplastic effects, which are tuned not only by the peptide sequence but also by the specific structural features of the lipid moiety, such as its chain length, degree of unsaturation, and methyl branching [38, 39, 40].

Prenyl groups confer membrane interactions distinct from those of linear fatty acyl chains, as their branched, unsaturated structures restrict conformational freedom and alter packing within the lipid bilayer [41]. Consequently, the architecture of the attached lipid serves as a critical determinant of lipidation outcomes and a key design parameter in protein and peptide engineering. This review specifically focuses on prenylation, introducing three major classes of prenyltransferases: eukaryotic protein prenyltransferases (PTs) and two bacterial enzyme families that modify peptide substrates [42]. The bacterial *trans*‐isoprenyl diphosphate synthase (*trans*‐IDS)‐like prenyltransferases are particularly emphasized as emerging tools that access longer‐chain donors and enable regioselective tryptophan modification, thereby expanding the available chemical space [26, 43]. We compare the physicochemical properties and biological outcomes of prenyl groups, including both canonical cysteine *S*‐prenylation and tryptophan C‐prenylation, with those of fatty acyl modifications through experimental data and in silico analyses. Finally, we survey applications ranging from antimicrobial peptide (AMP) engineering and high‐throughput screening to emerging areas such as lipidation‐tuned liquid–liquid phase separation (LLPS), concluding with practical directions for the future of data‐driven peptide design.

## 2 Protein/Peptide Prenyltransferases

### 2.1 Biosynthesis of Prenyl Diphosphate Donors

**FIGURE 2:**
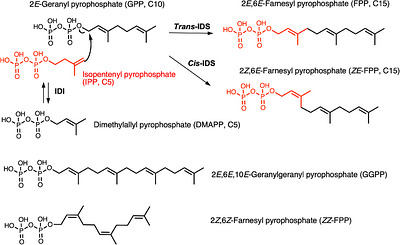
Biosynthesis and structural diversity of prenyl diphosphate donors. The five‐carbon unit IPP is isomerized to DMAPP by IDI, followed by chain elongation catalyzed by *trans*‐IDSs to yield all‐*E* configured donors, including GPP (C10), FPP (C15), and GGPP (C20). In contrast, *cis*‐IDSs catalyze the formation of *Z*‐containing isomers, such as *ZE*‐FPP and *ZZ*‐FPP.

A brief overview of prenyl diphosphate donor biosynthesis provides the essential context for discussing prenylation chemistry and the mechanisms of various prenyltransferases. Prenyl diphosphates are synthesized through the head‐to‐tail condensation of five‐carbon (C5) isopentenyl diphosphate (IPP) units [44] (Figure 2). IPP is interconverted with its isomer, dimethylallyl diphosphate (DMAPP, C5), by IPP isomerase (IDI) [45]. *Trans*‐isoprenyl diphosphate synthases (*trans*‐IDSs) then catalyze sequential IPP additions to allylic diphosphates, yielding geranyl diphosphate (GPP, C10), farnesyl diphosphate (FPP, C15), and geranylgeranyl diphosphate (GGPP, C20) [44]. These isoprenoid chains typically adopt the *trans* (*E*) configuration. Certain *trans*‐IDSs extend these chains further to produce longer‐chain diphosphates (≥ C25), such as geranylfarnesyl (GFPP), solanesyl, and polyprenyl diphosphates [46]. In contrast, *cis*‐isoprenyl diphosphate synthases (*cis*‐IDSs), which are evolutionarily distinct from *trans*‐IDSs, generate *cis* (*Z*)‐configured isoprenoid chains and contribute to the biosynthesis of long‐chain isoprenoids such as undecaprenol diphosphate (C55) and dolichol phosphate (C55–C100) [47, 48]. Furthermore, *cis*‐configured donors, such as (2*Z*,6*Z*)‐farnesyl diphosphate (*ZZ*‐FPP, C15) synthesized via neryl diphosphate elongation, are also available as enzymatic reagents [49] (Figure 2). These allylic diphosphates serve as the primary prenyl donors for the prenyltransferases detailed in the following sections.

### 2.2 Eukaryotic Prenyltransferases Install C15/C20 Prenyl Groups on Cysteine Residues

**FIGURE 3:**
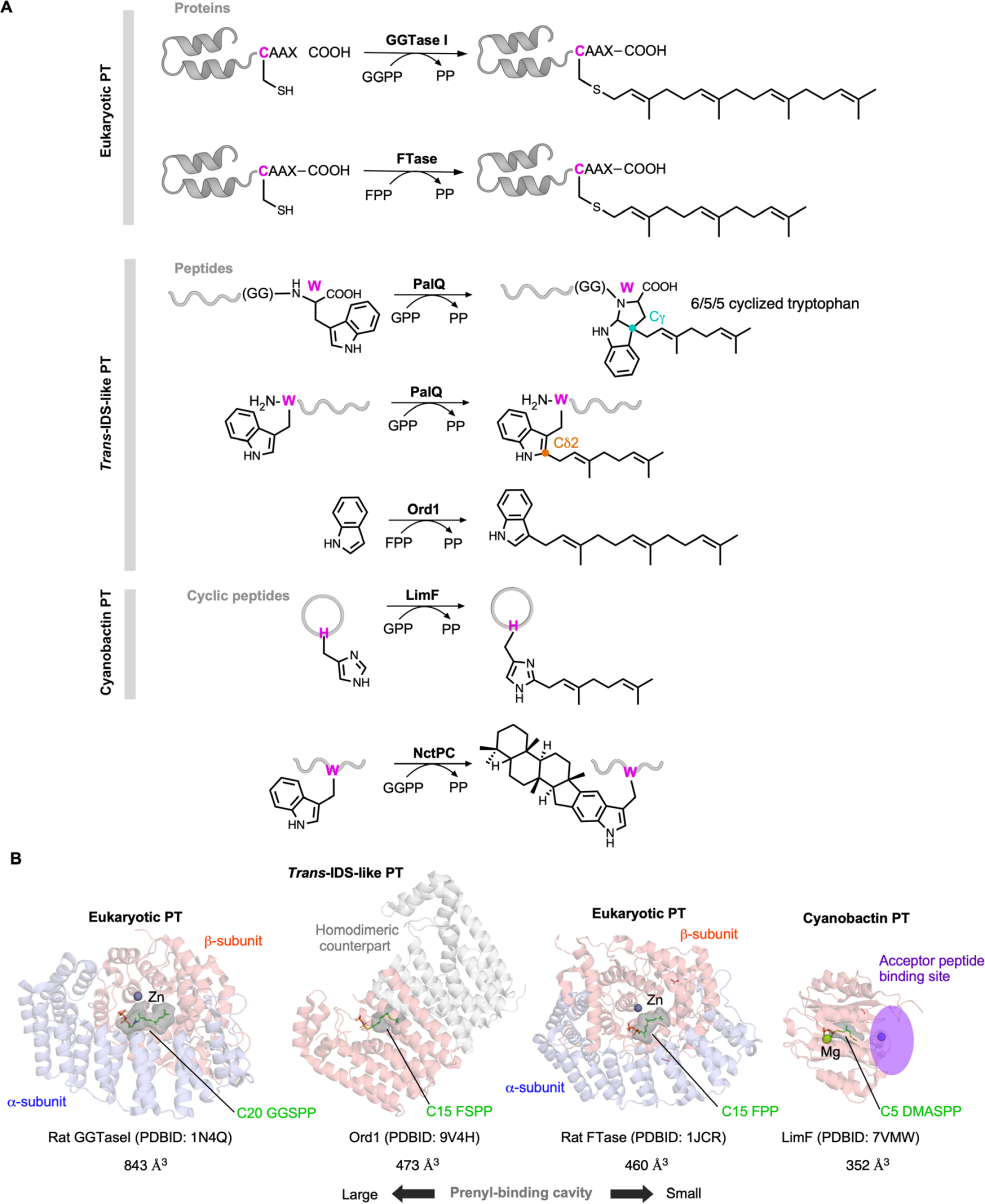
Three major classes of prenyltransferases. (A) Representative reactions catalyzed by eukaryotic, *trans*‐IDS‐like, and cyanobactin prenyltransferases. (B) Crystal structures of representative enzymes, with the prenyl‐donor binding cavities highlighted as grey surfaces. GGSPP, FSPP, and GSPP denote less‐reactive thio‐diphosphate analogues, featuring a sulfur atom in place of the bridging oxygen. Cavity volumes (Å^3^) were determined using CAVER 3.0 [57].

PTs are generally classified into three groups: eukaryotic PTs, cyanobactin PTs, and *trans*‐isoprenyl diphosphate synthase (*trans*‐IDS)‐like PTs (Figure 3A) [26, 42]. Eukaryotic PTs include farnesyltransferase (FTase), which attaches a farnesyl group, and several geranylgeranyltransferases (GGTases), including GGTase I, GGTase II, and GGTase III, which install geranylgeranyl groups [50]. These enzymes are heterodimers consisting of an α‐subunit and a β‐subunit, with their prenyl‐donor specificity dictated by the dimensions of a hydrophobic cavity within the β‐subunit (Figure 3B) [51]. For example, the W102βT mutation in the FTase β‐subunit enlarges this binding pocket, shifting its preference toward GGPP over the natural donor FPP [52]. Furthermore, eukaryotic PTs also exhibit substrate promiscuity toward nonnatural prenyl diphosphate analogues, facilitating site‐specific protein labeling with fluorophores or bioorthogonal functional groups [53]. Protein‐engineering efforts have further expanded this utility by generating variants with enhanced activity toward these synthetic analogues [54, 55, 56].

Eukaryotic PTs recognize specific *C*‐terminal motifs within their protein substrates. Both FTase and GGTase I target the CaaX motif, where *C* represents the target cysteine and “a” denotes an aliphatic residue. Their distinct preferences for the *X* residue determine whether the cysteine undergoes farnesylation or geranylgeranylation [58]. In contrast, GGTase II requires an additional Rab escort protein (REP) to modify *C*‐terminal sequences containing two cysteines, such as CXCX, CCXX, or XXCC, typically catalyzing dual geranylgeranylations [59]. This double modification is essential for accurate protein localization to specific membranes [60]. Furthermore, GGTase III reportedly geranylgeranylates specific substrates, including FBXL2 and Ykt6 [61]. A fundamental question in this field is why eukaryotic PTs exhibit such high selectivity for cysteine as the prenyl acceptor. These enzymes share a conserved Zn^2+^ ion in their active sites, which coordinates the cysteine thiol and lowers its p*K*a. This interaction generates a highly nucleophilic zinc–thiolate intermediate, thereby promoting efficient C─S bond formation [62].

### 2.3 Cyanobactin Prenyltransferases Install Short‐Chain Prenyl Groups on Diverse Residues

Cyanobactin prenyltransferases (PTs), also known as F‐family PTs, adopt an αββα barrel fold [15, 37]. This fold is shared with ABBA‐type aromatic PTs, although the sequence similarity between the two groups is low [15, 48]. Compared to canonical ABBA enzymes, cyanobactin PTs typically possess a more accessible active‐site pocket, largely due to the absence of certain secondary‐structure elements that otherwise enclose the catalytic cavity (Figure 3B). This open architecture is thought to accommodate bulky peptide substrates, particularly macrocyclic peptides, by facilitating access to a catalytic center where one or two Mg^2^^+^ ions coordinate the pyrophosphate moiety of the prenyl donor. While cyanobactin PTs generally utilize short‐chain donors such as C5‐DMAPP or C10‐GPP, likely because of steric competition with the peptide acceptor, their donor selectivity can be reprogrammed through mutagenesis. For example, engineered variants of LimF and PagF reportedly enable the transfer of C15 farnesyl groups [63]. In addition to canonical forward prenylation (at C1 of the prenyl group), these enzymes can catalyze reverse prenylation (at C3) [29, 32, 34, 64] (Figure 2).

Recently, NctPC was reported as an αββα barrel fold PT with low sequence similarity to known cyanobactin PTs [26, 42]. NctPC is a bifunctional fusion enzyme comprising a prenyltransferase (P) domain and a cyclase (C) domain, with the latter resembling the monodomain terpene cyclase MstE [65]. Of particular interest, NctPC transfers a C20 geranylgeranyl group to tryptophan, followed by subsequent cyclization by the C domain (Figure 3A).

Although the mechanistic details remain limited, these reactions are hypothesized to proceed via an SN1‐like dissociative pathway, by analogy to ABBA‐type enzymes such as dimethylallyltryptophan synthase [66]. In this model, the departure of pyrophosphate generates an allylic carbocation stabilized by metal coordination, with cationic character distributed across C1 and C3. Nucleophilic attack at C1 or C3 yields forward or reverse prenylation, respectively. Such a dissociative mechanism may explain the prenylation of relatively weak nucleophilic sites [67], although further experimental validation is required.

### 2.4 *Trans*‐IDS‐Like Prenyltransferases Install Longer‐Chain Prenyl Groups on Tryptophan Residues

*Trans*‐IDS‐like PTs include ComQ (InterPro: IPR033965) [21], the P domain of ChrPC [26], and PalQ [43] (InterPro entries not yet assigned for the latter two). As these enzymes belong to the isoprenoid synthase domain superfamily (InterPro: IPR008949), they are thought to share an evolutionary origin with canonical *trans*‐IDSs (InterPro: IPR000092) [26, 42, 48, 68]. Many *trans*‐IDS‐like PTs form homodimers, a structural feature shared with numerous *trans*‐IDSs [43] (Figure 3B). Compared with cyanobactin PTs, *trans*‐IDS‐like PTs tend to install longer prenyl groups, typically ranging from C5 to C20, and some members can even accept *cis*‐configured donors such as *ZZ*‐FPP [68]. AlphaFold models suggest that these enzymes possess an overall architecture similar to that of Ord1, a PT that catalyzes the C15 farnesylation of free tryptophan [43, 69]. The crystal structure of Ord1 revealed that the prenyl‐binding cavity is oriented toward the homodimer interface [43] (Figure 3B). This unique spatial arrangement alleviates steric hindrance, thereby allowing the accommodation of longer prenyl chains. In PalQ, the wild‐type enzyme efficiently utilizes C10 donors, while a quadruple‐alanine mutant with an enlarged cavity expands the scope to include C20 GGPP [68].

To date, all characterized *trans*‐IDS‐like PTs specifically target tryptophan residues [42]. ComQ catalyzes forward the prenylation of an internal tryptophan, followed by an intramolecular cyclization that yields a 6/5/5 cyclized tryptophan motif [21, 70, 71]. The P domain of ChrPC similarly modifies the internal tryptophan residue with C10–C20 donors, while the C domain facilitates the subsequent cyclization of the prenyl group [26]. In contrast, PalQ exhibits position‐dependent reactivity by geranylating an *N*‐terminal tryptophan at the *C*_δ2_ position and prenylating a *C*‐terminal tryptophan at the *C*_γ_ position [43, 68]. The latter modification also results in a 6/5/5 cyclized motif (Figure 3A). PalQ requires strict recognition of the peptide terminus, as *C*‐terminal extensions beyond tryptophan abolish its activity. Docking simulations suggested that productive catalysis requires the deep insertion of the acceptor tryptophan into the active site, a geometry that is favored when the residue is terminal [43, 68]. Notably, glycine substitutions at the −2 to +2 positions flanking an internal tryptophan allowed detectable, albeit low, prenylation activity. Substituting tryptophan with other potentially nucleophilic residues likewise abolished the activity, indicating a strong preference for tryptophan [43].

**FIGURE 4:**
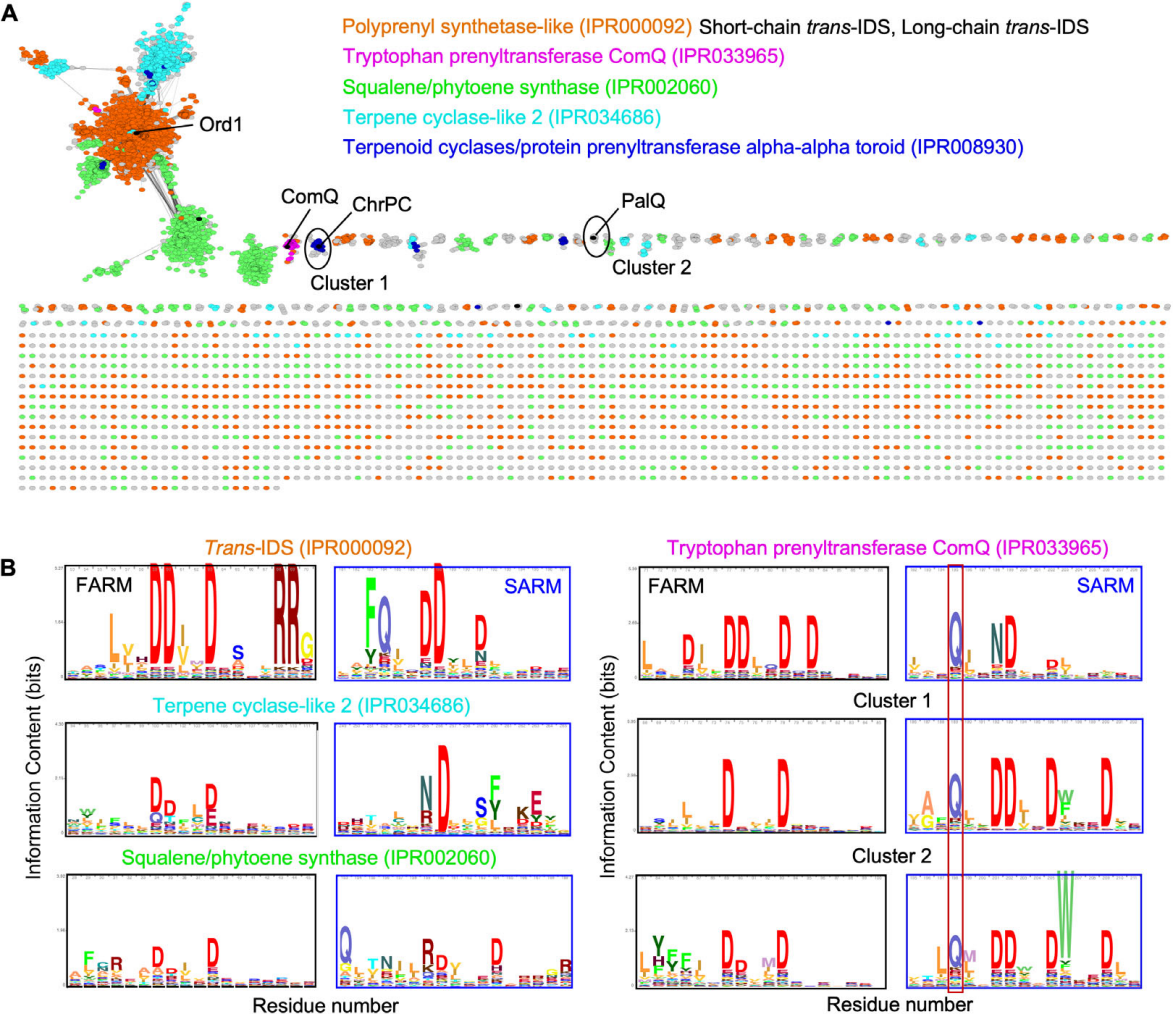
(A) Sequence similarity network (SSN) of the isoprenoid synthase domain superfamily (IPR008949). Amino acid sequences were retrieved from the UniRef50 database and processed through the EFI‐EST pipeline [76], with the resulting network visualized in Cytoscape [77]. Nodes were clustered using an alignment score threshold of 30, which corresponds to approximately 32% sequence identity. Nodes annotated as *trans*‐isoprenyl diphosphate synthases (*trans*‐IDS; IPR000092), terpene cyclase‐like 2 enzymes (IPR034686), squalene/phytoene synthases (IPR002060), and the tryptophan prenyltransferase ComQ family (IPR033965) are highlighted in orange, cyan, green, and magenta, respectively. (B) Sequence logos for the first and second aspartate‐rich motifs, known as FARM and SARM, were generated using WebLogo [78]. In these logos, higher information content measured in bits indicates greater residue conservation. Putative glutamine residues involved in tryptophan recognition are highlighted with red boxes.

To investigate how these enzymes acquired tryptophan selectivity, we performed a sequence‐similarity network (SSN) analysis to visualize the evolutionary divergence within the superfamily (Figure 4A). We first assembled representative sequence sets for *trans*‐isoprenyl diphosphate synthases, terpene cyclase‐like 2 enzymes, and squalene/phytoene synthases. Additionally, we curated sequence sets from two SSN‐defined clusters of *trans*‐IDS‐like PTs, where one cluster contained ChrPC (cluster 1) and the other contained PalQ (cluster 2). We then compared the sequence conservation around two motifs that are critical for catalysis: specifically, the first and second aspartate‐rich motifs, known as FARM and SARM, respectively (Figure 4B). In canonical *trans*‐IDSs, the FARM coordinates the allylic donor while the SARM positions the IPP substrate [72, 73]. In *trans*‐IDS‐like PTs, the SARM motif has been repurposed for the recognition of the tryptophan moiety rather than the IPP [74, 75]. These enzymes and the related enzyme Ord1 share a conserved glutamine residue located immediately upstream of the SARM [69]. AlphaFold models and mutational studies suggest that this glutamine, together with the first aspartate of the SARM, forms a local hydrogen‐bonding network positioned near the indole N─H of tryptophan [43, 69]. These results implicate a conserved, glutamine‐centered hydrogen‐bond network as a key determinant of tryptophan recognition.

## 3 Physicochemical Properties of Isoprenylation

### 3.1 Lipid Rafts and Membrane Phase Behavior

**FIGURE 5:**
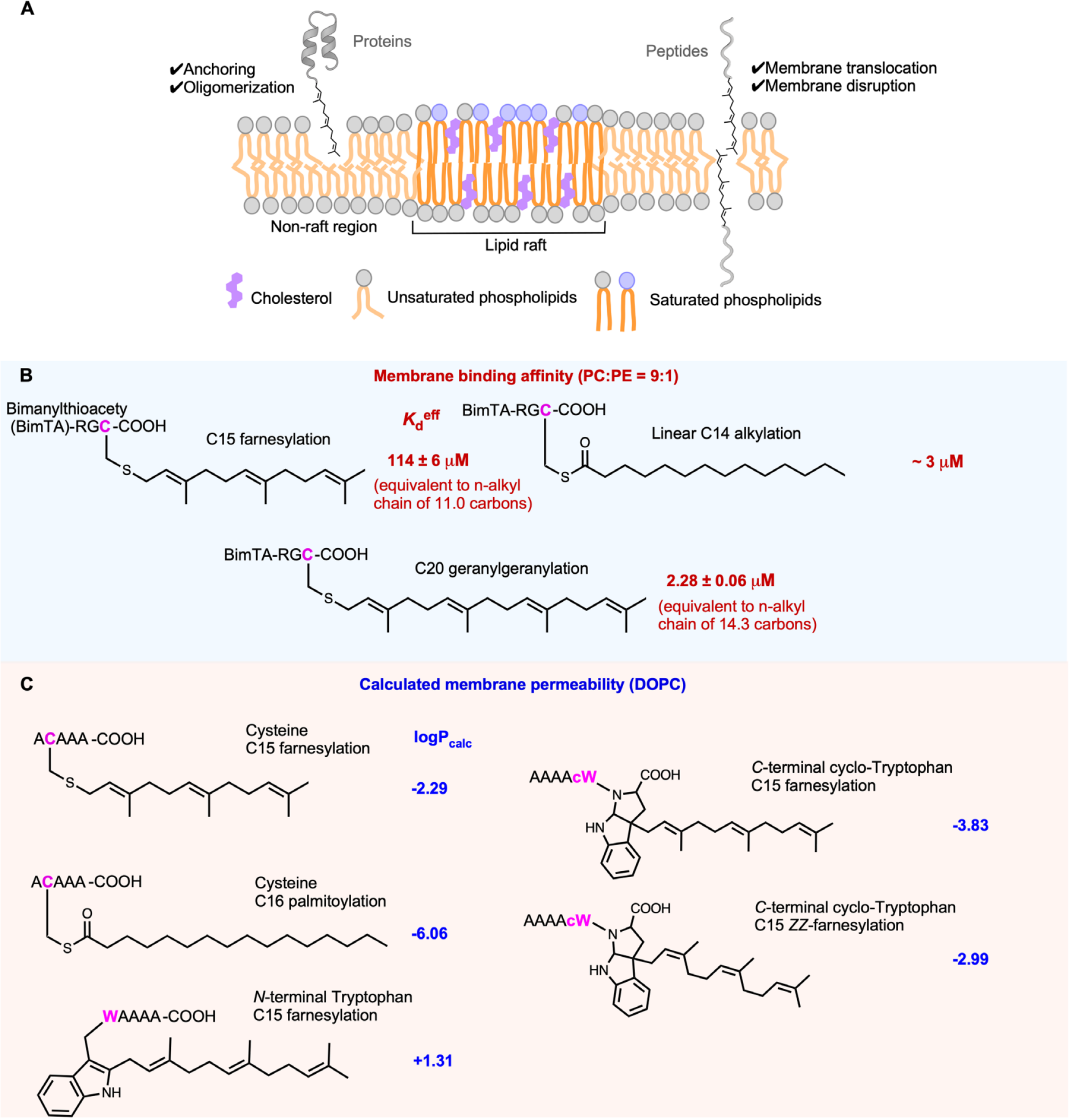
Physicochemical consequences of prenyl lipid anchors. (A) Schematic illustration of membrane lateral heterogeneity showing raft and nonraft regions. Prenylated proteins are often enriched in nonraft regions, where prenylation can support membrane anchoring and, in some instances, clustering. For short and highly charged peptides, prenylation can also promote membrane penetration and disruption rather than acting solely as an anchor. (B) Membrane‐binding affinities of a model peptide, Bim‐TA–RGC–COOH, bearing different cysteine‐linked lipid groups. These values were measured using neutral PC/PE (9:1) vesicles through fluorescence enhancement. Effective dissociation constants (*K*_d_^eff^) are provided for linear C14 n‐alkylation, C15 farnesylation, and C20 geranylgeranylation. Data are reproduced from [6]. (C) Predicted membrane permeabilities of representative prenylated motifs calculated with the CELLPM server (https://cellpm.org/cellpm_server_cgopm) in a DOPC bilayer, reported as logP_calc_. In this framework, a higher logP_calc_ indicates higher predicted permeability, and values above −5 are consistent with more efficient bilayer penetration.

Biological membranes consist of a diverse array of lipid species, where preferential lipid–lipid interactions drive lateral heterogeneity and, in certain contexts, promote LLPS [79, 80]. In the plasma membrane, lipid rafts are often described as more tightly packed and more ordered nanoscale domains that are enriched in saturated phospholipids, sphingolipids or glycolipids, and cholesterol (Figure 5A). These domains are relatively resistant to detergent extraction and are widely regarded as organizing platforms for membrane‐associated signaling [79, 81]. In contrast, nonraft regions are generally more fluid and enriched in unsaturated lipids. Similar phase behavior can be reproduced in multicomponent model membranes, where raft‐like domains correspond to the liquid‐ordered (*L*_o_) phase while nonraft regions correspond to the liquid‐disordered (*L*_d_) phase [82].

Protein lipidation contributes to membrane targeting and can bias partitioning between membrane domains [5]. GPI‐anchored and palmitoylated proteins often associate with raft domains, whereas prenylated proteins tend to be enriched in nonraft regions. Some prenylated proteins, such as singly farnesylated K‐Ras4B, can partition between raft and nonraft domains [83]. When K‐Ras4B is recruited to raft domains, the farnesyl chain packs poorly with the surrounding ordered lipids, inducing a hydrophobic mismatch and less stable membrane insertion. Such destabilization of the lipid anchor has been proposed to promote protein clustering or oligomerization [83, 84].

### 3.2 Entropic Penalties and Hydrophobic Mismatches Modulate Prenyl–Membrane Interactions

To experimentally assess how the chemical nature of the lipid anchor influences membrane binding, Silvius and coworkers quantitatively compared the membrane affinities of an RGC tripeptide bearing cysteine‐linked n‐alkyl or prenyl groups [6]. The peptides were labeled at the *N*‐terminus with an *S*‐bimanylthioacetyl (Bim‐TA) group, and the fluorescence enhancement upon vesicle binding was used to derive effective dissociation constants (*K*_d_^eff^). For PC/PE (9:1) vesicles, which are zwitterionic, net‐neutral bilayers that primarily report hydrophobic insertion, C14 alkylation gave a *K*_d_^eff^ = 3 µM, whereas C15 farnesylation and C20 geranylgeranylation yielded 114 and 2.28 µM, respectively (Figure 5B). Indeed, the *K*_d_^eff^ for C15 farnesylation corresponds to that of an n‐alkyl chain of approximately 11 carbons, indicating relatively weak membrane affinity despite a comparable carbon count. The measured affinity exhibited a pronounced dependence on membrane composition. For C15 farnesylation, *K*_d_^eff^ increased to 319 µM in cholesterol‐containing ePC/tPE/chol (9:1:6.7) vesicles, which represent a more condensed and ordered bilayer environment. Conversely, it decreased to 67.4 µM in tPE/ePC/DOPS (6:2:2) vesicles, an anionic, cytoplasmic‐leaflet–mimetic membrane containing the unsaturated lipid DOPS. These measurements demonstrate that the membrane‐binding contribution of a prenyl anchor depends on both the anchor chemistry and the lipid environment.

Two physical factors likely contribute to why prenyl anchors can confer weaker apparent membrane affinity than linear n‐alkyl chains. First, membrane insertion entails a significant entropic penalty, stemming from the restricted conformational freedom of the branched lipid chain. Reuther and coworkers used ^2^H and ^13^C solid‐state NMR to analyze doubly lipidated N‐Ras, featuring both palmitoyl and farnesyl groups, in DMPC vesicles, which are single‐component zwitterionic PC bilayers with saturated C14 chains [85]. From the ^2^H order parameters, they quantified the dynamics of the palmitoyl chain, which remained relatively mobile, especially near the terminal segment. Consistent with this, the protein backbone near the palmitoylation site showed greater mobility than that near the farnesylation site, as judged from the ^1^H–^13^C order parameters for the Cα positions at Cys181 (palmitoylated) and Cys186 (farnesylated). Although these observations come from a doubly lipidated context, they suggest that palmitoyl anchoring carries a smaller entropic cost than farnesyl anchoring.

Second, hydrophobic mismatches can further penalize prenyl insertion in ordered membranes. Zhang and coworkers performed all‐atom MD simulations of a 12‐residue peptide bearing either a palmitoyl or a farnesyl modification in an inner‐leaflet raft model membrane [41]. Under ordered (*L*_o_‐like) conditions at 300 K, farnesyl chains adopted larger tilt angles and higher lateral diffusion coefficients than palmitoyl chains, which is consistent with poorer packing in an ordered environment. Farnesyl chains also contacted unsaturated lipid chains more frequently, whereas palmitoyl chains were more often associated with saturated lipid tails and cholesterol. Under disordered (*L*_d_‐like) conditions at 330 K, these differences were much less pronounced. Taken together, the NMR and MD data converge on a common picture: even in liquid‐ordered bilayers that retain substantial segmental motion, farnesyl groups incur larger entropic and mismatch/packing penalties than palmitoyl anchors, consistent with weaker membrane associations under *L*_o_‐like conditions.

### 3.3 Prenylation Chemistry Beyond Cysteine and Its Dynamic Implications for Membrane Interactions

The differences in chemical structures and physicochemical properties between cysteine *S*‐palmitoylation and cysteine *S*‐prenylation can influence how lipidated proteins interact with membranes [7]. Parameters such as chain length, unsaturation pattern and geometry, and methyl branching, as well as the conformational rigidity and steric bulk of isoprenoid lipids are all expected to modulate membrane affinity and domain partitioning. Mechanistic insight in this area has been obtained mainly for eukaryotic cysteine *S*‐prenylation, whereas the membrane‐binding consequences of prenylation at non‐cysteine sites remain much less well defined.

*Trans*‐IDS‐like prenyltransferases expand the prenylation‐derived chemical space by catalyzing tryptophan *C*_γ_ prenylation, yielding a cyclized tryptophan with a 6/5/5‐fused ring system. The formation of the additional five‐membered pyrrolidine ring directly alters the local peptide‐backbone architecture and introduces new modes of conformational dynamics, including prolyl‐like *cis–trans* isomerization of the adjacent amide bond and ring puckering [43, 71]. The intrinsic timescale of prolyl‐like *cis–trans* isomerization, typically around 10^−3^ s^−^^1^, is likely much slower than the subsecond‐to‐second dissociation of prenyl lipids from membranes [7, 86]. This separation of timescales suggests that isomerization is kinetically decoupled from individual binding or unbinding events, yet it may still contribute to the apparent kinetics by introducing slowly interconverting states with distinct membrane affinities [87]. Continued methodological development to install these non–*S*‐prenylation motifs into proteins, together with systematic experimental measurements of membrane binding, will be important for establishing the general principles underlying how prenylation chemistry and dynamics beyond cysteine modulate membrane association.

### 3.4 Tryptophan Prenylation as a Bioorthogonal Strategy

**FIGURE 6:**
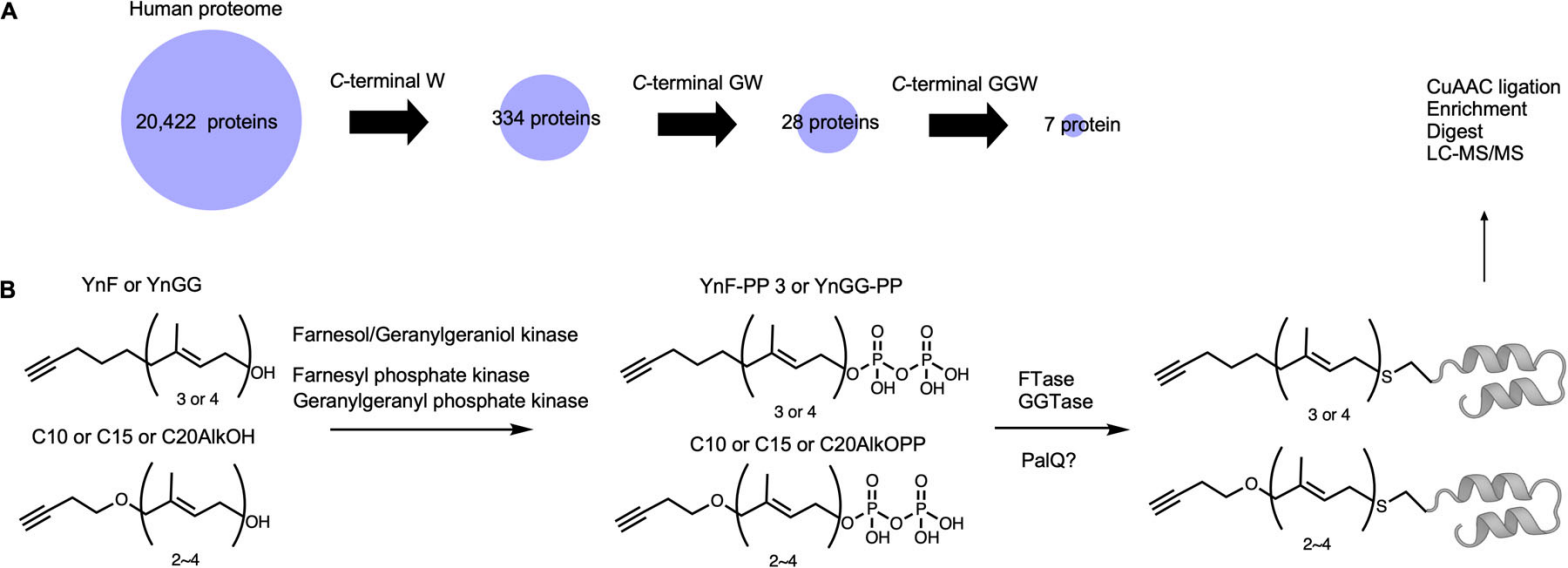
Bioorthogonal potential and labeling applications of PalQ‐mediated tryptophan prenylation. (A) Bioinformatic filtering of the human proteome (20,422 total proteins) for *C*‐terminal W, GW, or GGW motifs. (B) Conceptual workflow for the metabolic labeling and site‐selective functionalization of proteins using clickable, alkyne‐containing isoprenoid analogues as bioorthogonal prenyl donors.

The inherent scarcity of tryptophan in the proteome positions Trp‐directed chemistry as an attractive strategy for highly selective protein labeling [88, 89]. Analysis of the human UniProtKB/Swiss‐Prot proteome (Taxonomy ID 9606) reveals that among its 20,422 protein entries, only 334 possess a *C*‐terminal tryptophan (W), representing potential substrates for the *C*‐terminal Trp prenyltransferase PalQ (Figure 6A). The occurrence of more favorable PalQ recognition motifs [68] is even more restricted: only 28 proteins terminate with a GW motif, and 7 with a GGW motif. This limited endogenous background suggests that PalQ‐mediated prenylation could operate with high fidelity in eukaryotic cells, serving as a bioorthogonal platform for site‐selective protein functionalization and programmable membrane targeting.

The utility of such a system is further enhanced by the availability of synthetic prenyl donors. Alkyne‐containing isoprenoid alcohol analogues have been successfully utilized for the metabolic labeling of prenylated proteins in human cells [90, 91]. Following cellular uptake, these analogues are converted by endogenous kinases into their corresponding diphosphates, which are then utilized by native eukaryotic prenyltransferases (Figure 6B). Subsequent copper‐catalyzed azide–alkyne cycloaddition (CuAAC)‐mediated ligation to biotin or fluorescent dyes allows for the enrichment and visualization of over 100 prenylated targets [92]. While direct supplementation with diphosphate forms has been shown to improve the incorporation efficiency, these clickable isoprenoids may also serve as versatile alternative donors for PalQ. When integrated with heterologous PalQ expression, these probes provide a powerful route to functionalizing defined proteins within the complex environment of eukaryotic cells.

### 3.5 From Membrane Anchoring to Membrane Penetration

Experimentally, when a prenyl group is installed on short peptides rather than full‐length proteins, it can promote cellular entry through membrane penetration rather than simply serving as a membrane anchor. Wollack and coworkers synthesized a Cdc42‐derived peptide (Ac‐KCKKSRRC‐NH_2_) bearing a 5‐FAM label on the K1 side chain for detection [93]. C2 was protected with acetamidomethyl and *S*‐carbomethoxysulfenyl groups, while C8 was modified as an *S*‐geranylgeranyl (C20), *S*‐farnesyl (C15), or *S*‐methyl analogue. Since all‐*trans* prenyl alkenes are unstable under the acidic cleavage conditions of standard solid‐phase peptide synthesis, prenylation was performed in solution after resin cleavage. In HeLa cells, the *S*‐methyl control showed no detectable entry, whereas the prenylated peptides entered cells in a manner dependent on the prenyl chain length, with geranylgeranylation producing higher uptake than farnesylation. Follow‐up experiments further indicated that peptide length and net positive charge modulate entry efficiency [94]. Because the uptake was ATP‐independent and still occurred at 4°C, the authors argued against an endocytic mechanism and instead proposed a nonendosomal entry route.

To explore whether analogous non‐cysteine prenylation motifs might support bilayer translocation, we computationally evaluated their membrane permeability with the CELLPM server. Using the chemical structures in Figure 5C, we calculated the permeability coefficient (logP_calc_) for translocation across a DOPC bilayer. CELLPM applies the PPM 2.0 anisotropic‐solvent framework to calculate the position‐dependent transfer free‐energy profile, while optimizing the rotational orientation along the membrane translocation pathway, and then estimates the permeability using an inhomogeneous solubility–diffusion model. The transfer energetics include ASA‐dependent solvation, electrostatic contributions from dipoles/ions with depth‐dependent deionization, and a hydrophobic‐mismatch penalty, with additional penalties for bilayer deformation [95]. *S*‐farnesylation was predicted to confer substantially higher membrane permeability than *S*‐palmitoylation (Figure 5C). Tryptophan *C*‐prenylation motifs were also predicted to be more permeable than cysteine *S*‐palmitoylation motifs, which emphasizes that permeability depends strongly on the chemical nature of the lipid anchor. Among the tryptophan‐derived motifs examined here, predicted permeability increased in the order of *C*‐terminal Trp farnesylation, followed by *C*‐terminal cyclo‐Trp *ZZ*‐farnesylation and finally *N*‐terminal Trp farnesylation. Experimental measurements will be needed to validate these calculations and to establish how well such in silico permeability estimates translate to membrane translocation in cells and model bilayers.

Unlike small molecules, relatively large peptides often necessitate a high degree of conformational flexibility to achieve passive membrane permeability. In aqueous environments, the exposure of a relatively large polar surface area (PSA) is essential for maintaining solubility through hydration. Conversely, crossing the hydrophobic core of a lipid membrane favors a minimized PSA. Consequently, it has been proposed that optimal permeability relies on satisfying two distinct criteria: PSA_aq_ > 0.2×MW in water and PSA_np_ < 140 Å^2^ in nonpolar, membrane‐mimetic environments [96]. This minimization of exposed PSA is typically achieved by masking backbone amides through intramolecular hydrogen bonding, *N*‐methylation, or steric shielding by bulky nonpolar side chains. Interestingly, several membrane‐permeable macrocyclic peptides exhibit “chameleonic behavior”, characterized by a transition between two or more conformational states, often driven by peptide‐bond *cis*‐*trans* isomerization, to tune their PSAs in a solvent‐dependent manner [97]. This adaptability reconciles aqueous solubility with permeability in low‐dielectric environments. These principles are especially relevant to prenylated peptides, particularly those featuring proline‐like pyrrolidine rings that facilitate *cis*‐*trans* isomerization while simultaneously reducing the number of hydrogen bond donors. Detailed experimental characterizations, including NMR‐based solution structure determinations and conformational dynamics analyses, will be crucial for understanding how the interplay between structure and dynamics influences membrane permeability and the potential utility of these chemical handles for further modification.

## 4 Enhancing Peptide and Protein Function via Enzymatic Prenylation

### 4.1 Antimicrobial Activity

By exploiting the structural adaptability and dynamic conformational control discussed in the previous section, enzymatic prenylations are increasingly being leveraged to tune peptide bioactivity and to develop peptide‐based therapeutics. Many AMPs share key physicochemical features with cell‐penetrating peptides, including a propensity to adopt α‐helical conformations, a net cationic charge, and amphipathicity [98]. AMPs primarily act on bacterial membranes, which typically lack cholesterol but are enriched in distinctive components such as cardiolipin, lipid II, and, in Gram‐positive bacteria, lipoteichoic acid [99]. Proposed mechanisms include nonspecific membrane perturbation and destabilization, such as the carpet model, as well as pore formation pathways including toroidal and barrel‐stave pores [100]. It is worth noting that some AMPs also exhibit cytotoxicity toward eukaryotic cells, which may reflect analogous membrane‐perturbing activities when bacterial selectivity is compromised.

Naturally occurring lipopeptides are typically lipidated either at the *N* terminus through a fatty acyl moiety or at the *C* terminus through a fatty amine or alcohol [19, 101]. These lipid moieties often enhance antimicrobial potency by strengthening membrane association. Studies using synthetic membrane‐active peptides further suggest that optimal activity is generally achieved with intermediate‐length (C10–C12), unbranched, and unsaturated lipid tails, features that partially overlap with the physicochemical properties associated with prenylation [40].

**FIGURE 7:**
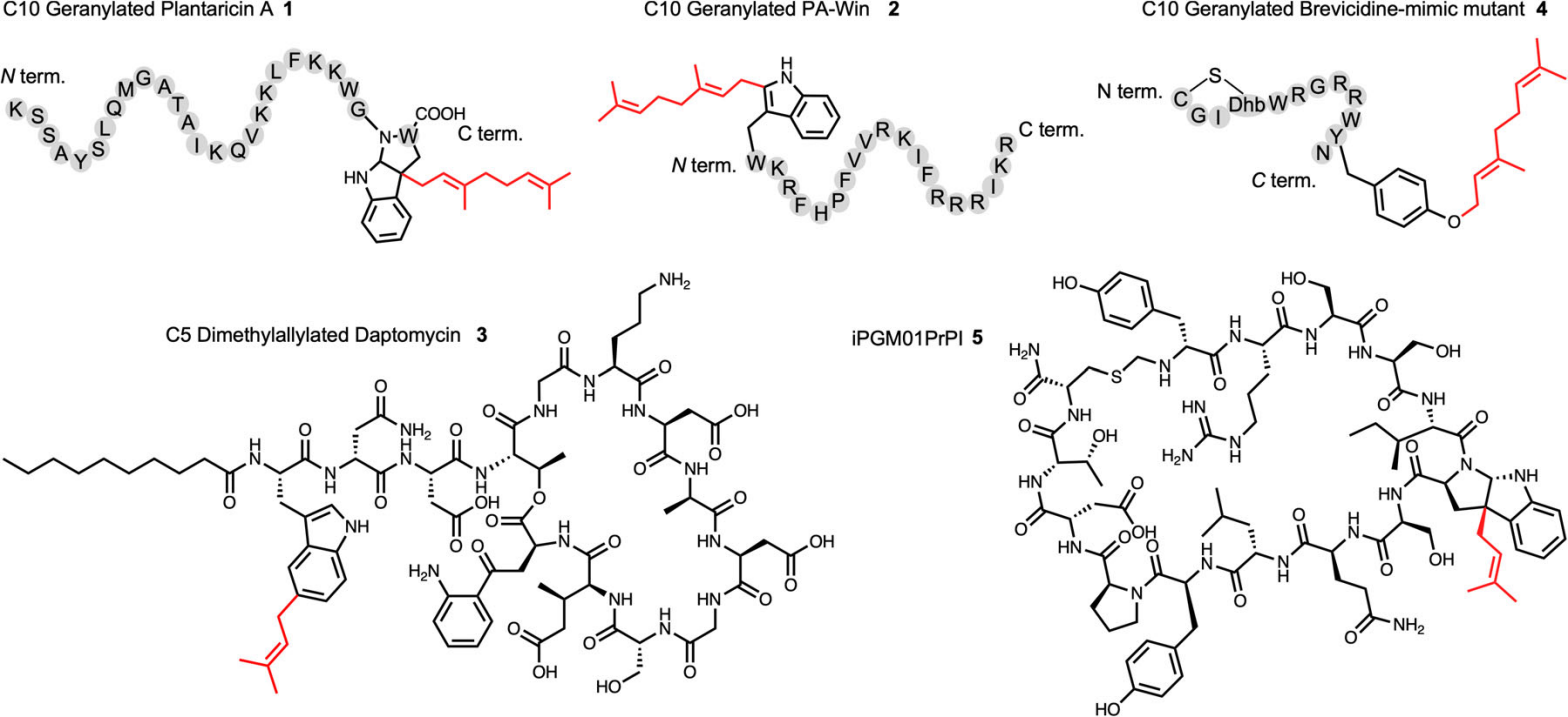
Enzymatic prenylation expands the structural diversification and functional enhancement of antimicrobial peptides (AMPs) **1–4** and the peptidic ligand **5**. Prenyl groups are highlighted in red.

Ozawa and coworkers applied the *trans*‐IDS‐like prenyltransferase PalQ to introduce *N*‐ and *C*‐terminal tryptophan prenylation across a panel of membrane‐active AMPs [68] (Figure 7, **1** and **2**). They found that a glycine residue immediately upstream of the prenyl‐acceptor tryptophan improves substrate recognition by PalQ. Across multiple peptides, antimicrobial activity was highest with C10 geranylation compared with C5 dimethylallylation or C15 farnesylation, mirroring the chain‐length optima often observed for acylated analogues [40, 102, 103]. NMR chemical‐shift perturbation experiments indicated that the prenyl group directly contributes to lipid interactions, and MD simulations supported the insertion of the prenyl moiety into bacterial membrane mimetics. Intriguingly, relative to C10 acylation, C10 prenylation produced a larger increase in antimicrobial potency. In line with the binding‐versus‐penetration considerations discussed above, these results are consistent with the idea that prenylation augments the membrane‐disruptive capacity of the peptides rather than merely enhancing their binding affinity. Moreover, C10 prenylation caused only modest cytotoxicity toward HL‐60 cells compared with C10 acylation. Because the enzymatic prenylation of highly cationic peptides with long‐chain prenyl donors often benefits from additives such as acetonitrile or salts, the authors designed a 23‐mutation PalQ variant using PROSS [104], an automated method for designing efficient and functionally diverse enzyme repertoires, which exhibited improved robustness under these reaction conditions.

Prenylation of AMPs has also been achieved using ABBA‐type aromatic prenyltransferases. Daptomycin is a cyclic lipopeptide that contains an *N*‐terminal tryptophan and is naturally *N*‐acylated with a C10 fatty chain [105]. Using indole prenyltransferases with distinct regioselectivities, PriB (*C*_δ2_ forward), FgaPT (*N*_ε1_ forward), and CdpNPT (*N*_ε1_ forward and *C*_γ_ reverse), C5 dimethylallylation was installed at different positions on the *N*‐terminal tryptophan [106]. The resulting prenylated daptomycin analogues showed a 6 to 10‐fold increase in antimicrobial activity without an apparent change in general cytotoxicity (Figure 7, **3**). However, these daptomycin derivatizations were performed using C5‐DMAPP as the prenyl donor, and longer‐chain prenyl donors were not evaluated on the daptomycin scaffold.

Related strategies have been extended to cyanobacterial tyrosine prenyltransferases [107]. Non‐ribosomal peptide‐mimicking precursor peptides were engineered to carry an *N*‐terminal Tyr‐*ψ* recognition motif, where *ψ* denotes any aliphatic or aromatic residue, enabling prenyl tail installation by PirF. The purified cyclic peptides were incubated with PirF and C10‐GPP, and geranylation was observed for substrates bearing an *N*‐terminal tyrosine as well as for those in which the tyrosine is positioned internally in a linear region. In this system, the geranylation substantially reduced the aqueous solubility, which limited biological evaluations of the purified products. Preliminary assessments using crude reaction mixtures showed that samples generated with active PirF exhibited stronger growth inhibition than non‐prenylated controls, which is consistent with geranylation contributing to enhanced antimicrobial efficacy (Figure 7, **4**).

### 4.2 Artificial Membrane Targeting via Prenylation Anchors

Artificial light‐controlled membrane targeting provides a powerful way to manipulate cellular processes with high spatial and temporal precision. A pioneering implementation uses blue light to promote reversible binding between two engineered protein modules, which drives the rapid translocation of a cytosolic fusion protein to a recruiter anchored at the plasma membrane [108]. In this design, the recruiter was constructed by fusing the CIB1‐derived binding region, CIBN, to enhanced GFP (EGFP) and appending a K‐Ras4B‐derived polybasic segment along with a CaaX prenylation motif (KKKKKKSKTKCVIM) at the *C*‐terminus. The CaaX motif serves as a canonical substrate for the endogenous mammalian FTase and is expected to undergo farnesylation within cells. In combination with the adjacent polybasic region, this modification supports targeting to the cytosolic face of the plasma membrane. The complementary cytosolic partner was generated by fusing mCherry to the photolyase homology region of cryptochrome **2** (CRY2PHR), which is a chromophore‐binding domain that binds flavin and pterin to undergo blue‐light‐dependent association with CIBN. Upon coexpression in mammalian cells, the CIBN–EGFP–CaaX recruiter localized to the plasma membrane, and blue light illumination triggered the rapid recruitment and translocation of CRY2PHR–mCherry from the cytosol to the membrane compartment. Building on this framework, subsequent studies fused CRY2PHR to the inositol 5‐phosphatase domain of OCRL, which enabled optogenetic control of phosphoinositide metabolism through the light‐driven recruitment of the enzyme to the plasma membrane [109].

Non‐eukaryotic modes of prenylation, particularly when combined with other posttranslational lipidations such as reversible cysteine *S*‐palmitoylation and irreversible *N*‐terminal myristoylation, are likely to provide a versatile route to selective membrane targeting and microlocalization across organelles with distinct lipid compositions, including the plasma membrane, mitochondria, the Golgi apparatus, peroxisomes, and endosomal membranes [110, 111]. Importantly, membrane selectivity is not determined solely by the chemical structure of the lipid moiety. Local sequence contexts around the lipidation site, together with downstream processing steps and partner interactions, can also function as critical determinants of subcellular localization [112].

### 4.3 Self‐Assembly of Prenylated Peptides and Proteins

More recently, peptide and protein lipidation, including prenylation, has emerged as a versatile determinant of higher‐order assembly. These modifications drive behaviors that span LLPS in bulk solution and condensation on membrane surfaces [113, 114, 115, 116]. Because the lipid chemical structure is a key determinant of phase behavior in bilayer membranes, the nature of the appended lipid is likewise expected to modulate the LLPS propensity and condensate material properties in solution. Mechanistic insight has been drawn primarily from work on *N*‐myristoylated resilin‐like polypeptides (RLPs), which are synthetic intrinsically disordered proteins that undergo phase separation upon heating [115]. In these studies, myristoylation was introduced in an *Escherichia coli* coexpression system using *N*‐myristoyltransferase (NMT), and the resulting myristoylated RLPs exhibited an increased phase‐separation propensity. A plausible explanation is that lipidation expands an effective hydrophobic patch around the modification site and reduces local hydration, which strengthens hydrophobic intermolecular association and promotes higher‐order assembly.

By analogy, prenylation may also influence LLPS and condensate material properties. In an engineered *E. coli* platform, heterologously expressed elastin‐like polypeptides (ELPs) bearing a *C*‐terminal CaaX motif were produced together with a geranylgeranyl pyrophosphate synthase (GGS) and a PT, enabling one‐pot in vivo prenylation [113]. In the absence of GGS, the prenyltransferase predominantly installed a C15 farnesyl group from endogenous FPP. Coexpression of GGS increased the cellular GGPP pool and changed the modification toward C20 geranylgeranylation. Both farnesylation and geranylgeranylation shifted the onset of phase separation to lower temperatures relative to unmodified ELPs, and the magnitude of this shift depended on the prenyl chain length. Notably, geranylgeranylated ELPs exhibited a sharp micelle‐to‐coacervate transition upon heating, which is consistent with the idea that the more hydrophobic C20 geranylgeranyl group stabilizes and strengthens the higher‐order assembly of the ELP chains.

### 4.4 Integrating Enzymatic Prenylation Into Peptide Display and Selection Platforms

Enzymatic prenylation has also been integrated into high‐throughput peptide discovery platforms. Inoue and coworkers combined the cyanobactin prenyltransferase KgpF, which catalyzes tryptophan C5 dimethylallylation followed by a 5/5/6 cyclization, with the RaPID (Random nonstandard Peptides Integrated Discovery) system to construct and select thioether‐macrocyclic peptide (teMP) libraries [117]. This integrated strategy enabled the identification of pseudo‐natural prenylated teMPs that inhibit specific target enzymes. Significantly, evaluation of a representative hit demonstrated that the prenyl modification directly conferred improvements in both serum stability and cellular uptake (Figure 7, **5**). These findings highlight the potential of enzymatic posttranslational modification as a versatile tool for enhancing the pharmacological properties of peptide‐based drug candidates.

## 5 Summary and Outlook

The structures, physicochemical properties, and biological functions of proteins and peptides are largely encoded in their amino acid sequences. Consequently, protein and peptide engineering has advanced rapidly through protein language models for sequence representation, diffusion‐based models for 3D structure generation, and MPNN‐based inverse folding for sequence design [118, 119, 120, 121, 122, 123, 124]. Most current models, however, operate within the canonical 20 amino acid alphabet, which largely overlooks the energetic, structural, and functional perturbations imposed by posttranslational modifications. Transitioning beyond this sequence‐centric paradigm necessitates the integration of posttranslational modifications as discrete structural tokens within model architectures to capture how they modulate local conformational landscapes and emergent biological functions.

Early demonstrations already point in this direction [125, 126]. For example, recent models have encoded *N*‐terminal lipidation to predict behaviors such as self‐assembly and to guide AMP design. These models were trained on datasets comprising 106 lipidated peptides curated from antimicrobial databases alongside 204 newly synthesized variants. Nevertheless, these collections lacked the branched and unsaturated motifs characteristic of prenylation. Expanding such libraries to systematically include regioselective and geometrically diverse prenylated peptides will be essential for building lipidation‐aware, data‐driven design models that capture the unique physicochemical space occupied by isoprenoids.

Prenylation serves as a biophysical determinant distinct from linear fatty acylation linear fatty acylation, as isoprenoids introduce complex sterics and restricted conformational degrees of freedom that engage membranes through distinct physicochemical interaction modes. Alongside the well‐studied cyanobactin enzymes, *trans*‐IDS‐like prenyltransferases are emerging as versatile tools for expanding the donor scope to longer chains and geometric isomers while enabling regioselective tryptophan modification. Establishing prenylation as a programmable parameter requires integrated databases spanning chain length, geometry, and sequence context. Scalable and modular workflows for reproducible library generation will be essential to define the relationships necessary for rigorous, data‐driven design.

Although considerable progress has been made in identifying polypeptide prenyltransferases and elucidating their catalytic mechanisms, major gaps remain in our understanding of how prenylation can be used to rationally control membrane behavior. Predictive relationships among prenyl chain structure, modification site, and functional output have yet to be established. Moreover, the design principles derived from polypeptide prenyltransferases have not been sufficiently extended to prenyltransferases acting on other biomolecular scaffolds, including oligosaccharides and nucleic acids. Addressing these gaps will be important for better understanding prenylation and for applying it to molecular engineering and therapeutic design.

## Conflicts of Interest

The authors declare no conflicts of interest.

## Data Availability

The data that support the findings of this study are available from the corresponding author upon reasonable request.
